# Insights into the feeding of jellyfish polyps in wild and laboratory conditions: do experiments provide realistic estimates of natural functional rates?

**DOI:** 10.1007/s10750-024-05783-0

**Published:** 2025-03-14

**Authors:** Cathy H. Lucas, Danja P. Höhn, Clive N. Trueman

**Affiliations:** 1 Ocean and Earth Science, National Oceanography Centre Southampton, University of Southampton, European Way, Southampton, SO14 3ZH UK; 2 Centre of Environment Fisheries and Aquaculture Science (CEFAS), Pakefield Road, Lowestoft, NR33 0HT UK

**Keywords:** *Aurelia aurita*, Polyps, Stable isotopes, Natural diet, Experiments

## Abstract

Biotic and abiotic factors that affect the physiology and ecology of scyphozoan polyps are considered to be major drivers of jellyfish blooms, but are rarely studied under field conditions. Here, stable isotopes of carbon and nitrogen were used to investigate feeding ecology in *Aurelia aurita* polyps from the Beaulieu River, UK (50° 80′ 04.55″ N/1° 42′ 28.12″ W) in both winter and summer conditions, and compared to laboratory-maintained polyps fed *Artemia* nauplii at 6 and 20 °C, respectively. In natural conditions, the isotopic composition of *A. aurita* polyps indicated assimilation of nutrients derived from both benthic and pelagic food pathways, with seasonal switches between benthic-derived nutrients in winter and pelagic-derived nutrients in summer. In laboratory experiments, polyps assimilated *Artemia* food at 6 °C although metabolic processes were reduced, while at 20 °C, polyps starved as their increased metabolic costs could not be met from the *Artemia* food. Experiments on growth and asexual reproduction of *Artemia*-fed polyps of *A. aurita* may not reflect natural metabolic rates especially at higher temperatures (e.g. 20 °C), because these polyps are not extracting sufficient resources from their *Artemia* food to fuel the increased metabolic costs associated with high temperatures.

## Introduction

Humans have often identified jellyfish (herein considered to include pelagic cnidarians and ctenophores) as a nuisance due to interferences with tourism, fishing, aquaculture, and power production (Purcell et al., 2007; Graham et al., 2014; Lucas et al., 2014b). Interactions between humans and jellyfish have received attention because of increased and altered uses of marine regions (e.g. power stations, marine aquaculture, coastal construction), and the possibility that climate warming and fishing may alter marine ecosystem structure in favour of jellyfish (Purcell, 2012, but see Pitt et al., 2018). Nevertheless, jellyfish are ubiquitous and natural members of marine ecosystems and make a major contribution to the total biomass in the ocean (Lucas et al., 2014a). They act as both predators and prey, and have the potential to affect biogeochemical cycling (Luo et al., 2020) and organic flux to the seafloor (Lebrato et al., 2012; Sweetman et al., 2014).

However, many aspects of the trophic role of jellyfish remain poorly described (e.g. Stoltenberg et al., 2021), and until recently, it has been difficult to define accurately both jellyfish diets and the contribution of jellyfish as prey for other consumers. With the application of stable isotope and fatty acid analyses (Javidpour et al., 2016; Pengpeng et al., 2020; Schaub et al., 2021) and DNA metabarcoding of gastrovascular contents (Lamb et al., 2017; Damien-Serrano et al., 2022; Dischereit et al., 2024), we now realise that jellyfish are not trophic dead-ends, but food for a variety of marine predators including fish, sea birds, mammals, turtles, and invertebrates (reviewed by Hays et al., 2018).

In comparison with the well-studied medusa life stage, rather little is known about in situ polyp populations (but see Prieto et al., 2010; Holst, 2012; Hočevar et al., 2018; van Walraven et al., 2020; Rekstad et al., 2021). The polyp enables bloom formation by increasing the source (budding new polyps) and supply (by strobilating new ephyrae) of new recruits to the medusa population (Lucas & Dawson, 2014). Information about the functional biology of polyps, including their nutrition, and how this translates into somatic and asexual reproductive growth is required to help predict the abundance of medusa populations between years and locations, including bloom formation (Lucas et al., 2012; Gambill & Peck, 2014; Schnedler-Meyer et al., 2018).

Because of their small size and the difficulty of using methods such as gut content analysis and lab clearance rates, there is very little information about polyp diet, feeding rates or metabolism under natural conditions (but see Wang et al., 2015; Marques et al. 2021; Pengpeng et al., 2021). Unlike the seasonally occurring pelagic medusa, polyp populations are present year-round (Hočevar et al., 2018; D.P. Höhn, unpublished data), so will experience a wide range of temperature and food conditions. Jellyfish with a metagenic life cycle typically inhabit coastal seas and estuaries, where a wide variety of food sources such as zooplankton, bacteria, and terrestrial- or marine-derived detritus are available. While it is possible that during spring and summer polyps feed on micro- and meso-zooplankton such as ciliates, copepods, and nauplii (Kamiyama, 2011, 2013; Pengpeng et al., 2021), these food resources become scarce in the winter months in temperate regions, and alternative, benthic diet sources may be important (see Sun et al., 2021 for *Aurelia coerulea* in the southern Yellow Sea region, China).

By contrast, laboratory-reared polyps used for measurements of growth and asexual reproduction are considered to be carnivorous, like the medusa life stage, and are successfully and regularly maintained on cultures of *Artemia* nauplii (brine shrimp) (Schiariti et al., 2008; Pascual et al., 2014). It is therefore unclear whether these laboratory conditions provide realistic representations of polyp diets and metabolism in natural settings, particularly in seasonally dynamic environments.

Stable isotope analysis can identify proportional contributions of nutrient sources to the diet of organisms if the potential nutrient sources are isotopically distinct (Iverson et al., 2002; Post, 2002). Tissue stable isotope compositions reflect those of the assimilated diet, integrated over the timescales required for tissue growth or turnover, which in turn reflects rates of somatic growth and metabolism. In temperate environments, the isotopic composition of carbon (expressed as *δ* ^13^C values) differs markedly between terrestrial and aquatic primary production, providing a natural tracer for nutrient supply from pelagic production or from microbial reworking of sedimented terrestrial detritus (e.g. Grey & Jones, 2001; Junker & Cross, 2014). Variations in nitrogen isotope ratios (expressed as *δ*^15^N values) predominantly, but by no means exclusively, give information on the relative trophic level (Cabana & Rasmussen, 1996; Peterson, 1999). Stable isotope approaches have frequently been applied to study diet in medusae (D’Ambra et al., 2014; McKenzie et al., 2014; Fleming et al., 2015; Javidpour et al., 2016; Schaub et al., 2021), but far less to the ecology of the polyp stage (but see recent papers by Pengpeng et al., 2021; Gießler et al., 2023).

Here, stable isotope approaches were used to study trophic ecology of polyps of the common scyphomedusa *Aurelia aurita* in both field and laboratory conditions. Polyps of the same population were deployed experimentally on settling plates in wild and laboratory habitats. In the natural environment, potential food sources were collected and their isotopic composition analysed. Laboratory-held polyps were raised using standard protocols and fed a diet of *Artemia* nauplii. Polyps were observed throughout the deployment period and stable isotope analyses used to identify the nutrient sources supporting polyp growth in the wild, and to record the nutritional response of polyps to laboratory conditions.

## Materials and methods

### Field location

**Fig. 1:**
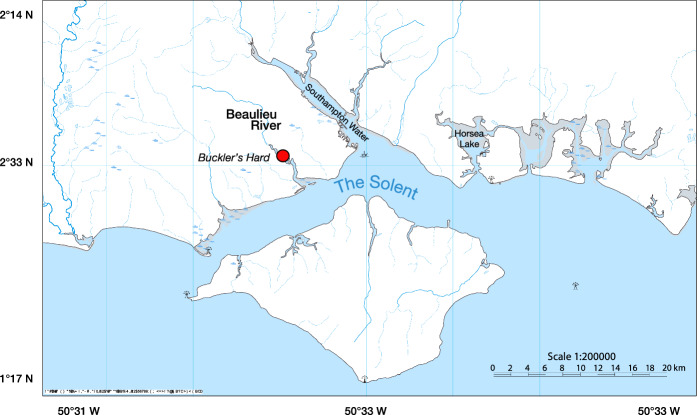
Study location at the Beaulieu River (Buckler’s Hard) in southern Britain. Degrees of latitude and longitude are shown on the y and x axes, respectively

Field experiments were conducted in the Beaulieu River, UK (50° 80′ 04.55″ N/1° 42′ 28.12″ W) (Fig. 1), an intertidal estuary that flows for 19 km south-westwards through the New Forest into the Solent estuarine system and drains 80 km^2^ of heathland and bog resulting in organic-rich water (Turner et al., 1998). The estuary is surrounded by saltmarshes and mudflats and has a tidal height of 4.4 m (Chen et al., 2011). Based on earlier, unpublished data (D.P. Höhn, unpublished data) from the Beaulieu River collected between 2014 and 2015, a seasonal pattern of zooplankton abundance is evident. Winter total zooplankton abundance is extremely low (32–118 individuals m^−3^) and is dominated by copepods. Peak zooplankton abundance varies inter-annually, with values between 3061 and 7704 individuals m^−3^ occurring in between April/May and July. Holoplankton, particularly copepods, are present throughout the year, with meroplankton, comprised of the larvae of polychaetes, molluscs, crustaceans, and fish, making significant contributions from March/April through to July. Regarding non-plankton sources of food, winter rainfall leads to considerable inputs from upstream of terrestrial and sediment-based detritus.

### Laboratory experiment

**Fig. 2:**
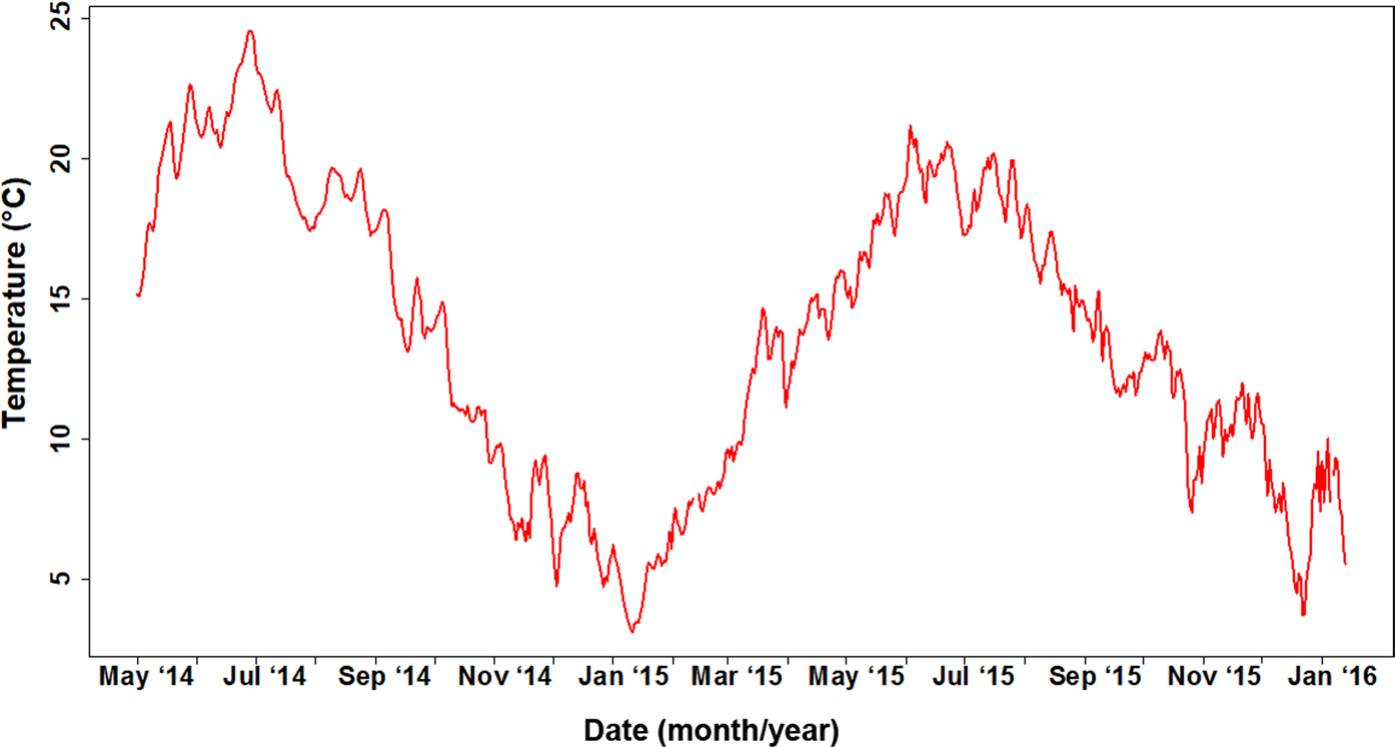
Daily mean surface water temperature (°C) at Buckler’s Hard in the Beaulieu River, from 28 May 2014 to 10 February 2016. Temperature data were logged continuously, with discrete measurements taken every 5 min

Fully-grown, healthy *Aurelia aurita* polyps of similar size were selected from the Beaulieu River stock culture, starved for 28 d, placed into 6 and 20 °C incubators and maintained in well-aerated filtered seawater with salinity of 31. Temperatures were chosen to reflect mean summer and winter temperatures in the Beaulieu River, which were 20.04 ± 1.91 °C between July and August 2015, and 6.35 ± 1.68 °C during January to February 2015 (Fig. 2). Polyps were fed daily, ad libitum*,* with *Artemia* nauplii (< 24 h old) to ensure polyps had more than sufficient food. Uneaten or dead nauplii were removed after 3 h to prevent degradation of water quality. It was noted that asexual reproduction in polyps was not observed. Water of the same temperature and salinity was replaced before each feeding event. For subsequent stable isotope analysis, individual *A. aurita* polyps were detached carefully over 42 d with tweezers from the settling plates, then washed and blotted dry on filter paper before being placed into pre-weighed tin capsules (pooled as 5 individuals per capsule) and frozen at −80 °C. Similarly, 13 whole *Artemia* were collected and prepared as above at the same time as the polyps.

### Field sampling

To quantify the extent that different food or nutrient sources support polyp growth in the field, benthic nutrient sources were characterised from detritus from terrestrial plants (e.g. *Spartina*, *Halimione*), wood litter (dried leaves, branches, bark), and sediment from the saltmarsh and river sediment from the Beaulieu. Pelagic nutrient sources were characterised from zooplankton and phytoplankton. Nutrient sources were sampled weekly between January and May 2016. Wood litter and plant materials were collected from the riverbank and placed into 5 ml Eppendorf cups on ice, then frozen at −80 °C. For riverine and saltmarsh sediment, the top 1 cm of the sediment surface was collected, filled into 5 ml Eppendorf cups on ice for transportation to the laboratory, where they were frozen at −80 °C. Zooplankton was collected with a 210 μm mesh plankton net, towed alongside a pontoon, with samples transferred into a 500 ml plankton bottle. Zooplankton (mainly crustacean copepods and larvae of decapods, polychaetes, barnacles, and molluscs) were selected under a stereomicroscope with fine forceps, washed and placed into tin capsules before being frozen at −80 °C. Zooplankton was not treated with acid prior stable isotope analysis as the effect of acidification on carbon isotope ratios is only significant for shelled molluscs and not for copepods (crustaceans), which comprised the majority of our zooplankton samples (Pomerleau et al., 2014), and the small volumes of plankton samples potentially risk sample loss during treatment. For the collection of phytoplankton, surface water was collected in 1000 ml plastic bottles. Seawater was filtered with a vacuum pump through a polycarbonate filter, and the remaining phytoplankton was scraped off the filter and placed immediately into pre-weighed tin capsules and frozen at −80 °C. Five replicates were prepared.

### Field experiment

*Aurelia aurita* polyps obtained from a stock culture originating from the Beaulieu River population were placed in the Beaulieu River at Buckler’s Hard marina on settling plates at about 1 m depth hanging from the pontoon in two phases for 42 d: during winter (from 17 February) and summer (from 20 July). Sub-samples of field-deployed polyps were collected weekly, simultaneous with the environmental sampling and were prepared as above. Special care was taken to remove any debris from the polyp.

### Sample processing

All fresh samples, i.e. polyps, nutrient sources including *Artemia*, were wet weighed then freeze-dried (Thermo Scientific Heto PowerDry LL33000) at − 50 °C for 12 h (for plant 24 h) to a constant weight, and subsequent dry weights (DW) were measured using a microbalance (Sartorius ME5). Wood litter, plant, and sediment samples were homogenised using a mortar and pestle and weighed into pre-weighed tin capsules. The isotopic composition of carbon (C) and nitrogen (N) (expressed as *δ*^13^C and *δ*^15^N values) was determined at the East Kilbride Node of the Natural Environment Research Council Life Science Mass Spectrometry Facility (UK) via continuous flow isotope ratio mass spectrometry using an ECS 4010 elemental analyser (Costech, Italy) interfaced with a Delta XP mass spectrometer (Thermo Electron, Germany). The standard deviation of multiple analyses of an internal gelatine standard was about 0.17 ‰ for *δ*^13^C and 0.15 ‰ *δ*^15^N. Stable isotope concentrations were expressed as *δ* notation as part per thousand (‰). Stable isotope ratios are reported as described by Bond & Hobson (2012).

### Data analysis

Data were analysed in R, version 3.0.2 (R Core Team, 2013). Generalised additive models (GAMs) were used to investigate the relationship between the isotopic composition of polyps (response variable) and time and temperature (explanatory variables) during the lab and field experiments (as described in Zuur et al., 2010). The freeze-dried weights of polyps at the start and end of the 42 days incubation at 6 and 20 °C, respectively, were compared using a Two-way ANOVA (factor temperature, starting weight, and weight at the end) after standard testing for normality and homogeneity of variance.

## Results

### Laboratory experiment

**Fig. 3:**
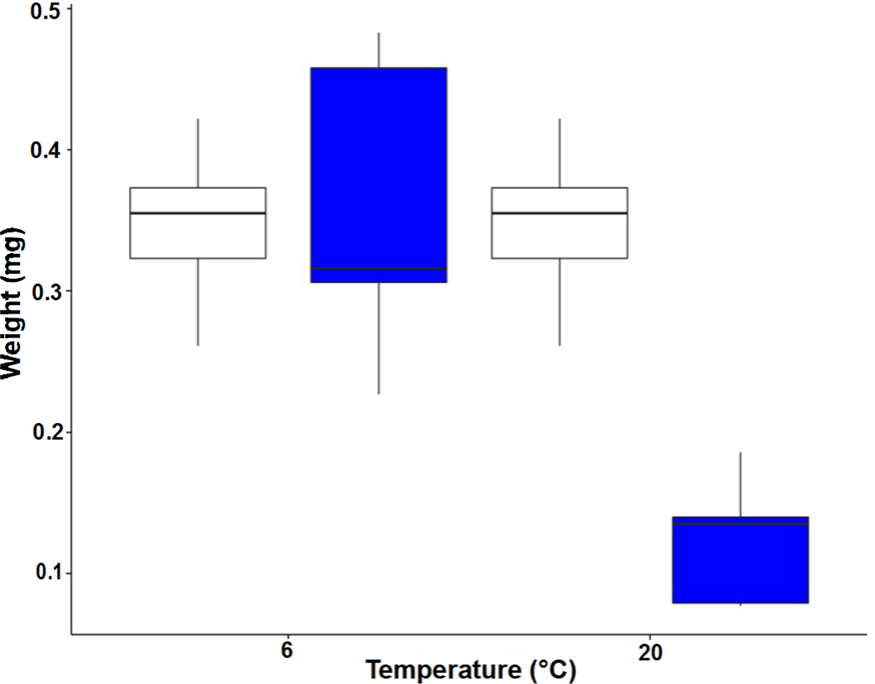
*Aurelia aurita* polyp individual dry weights shown as boxplots with starting weight (white) and end weight (blue) at 20 °C and 6 °C. The dry weight at 20 °C was 41% lower after 42 days (Two-way ANOVA, F(1,16) = 7.04, *P* < 0.05). *n* = 3 per condition

**Fig. 4:**
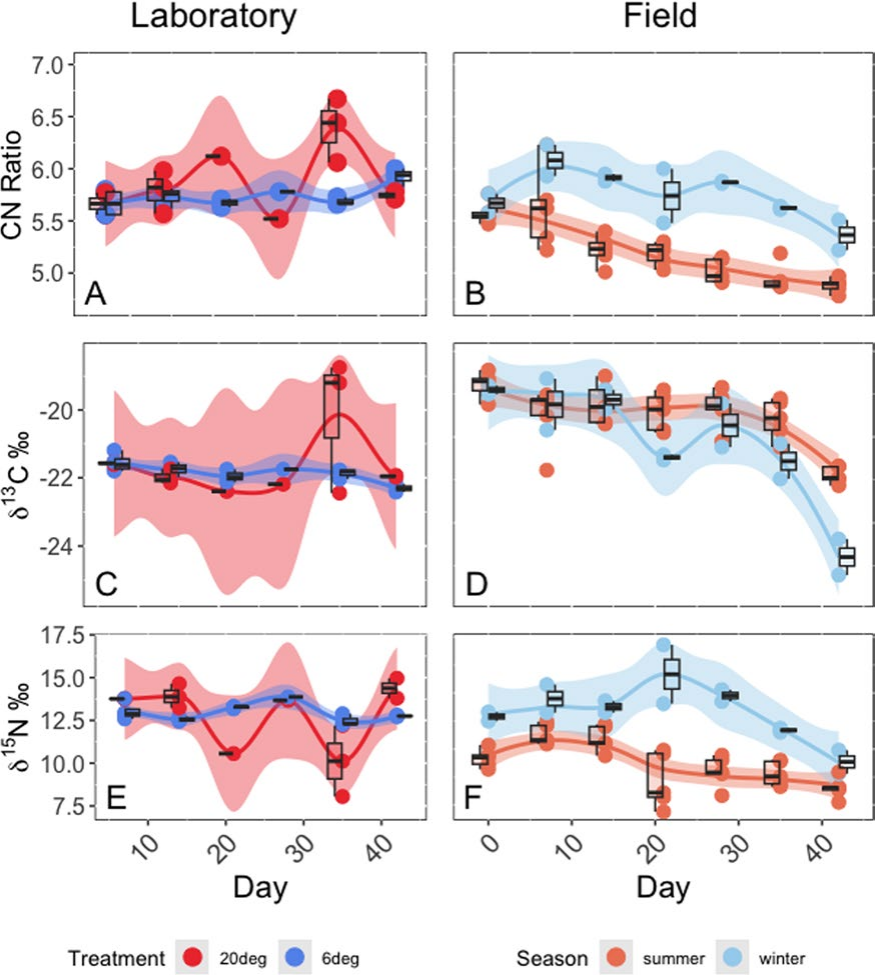
CN molar ratios (**A**,**B**), δ^13^C values (**C**,**D**) and δ^15^N values (**E**, **F**) observed in *Aurelia aurita* polyps over time under laboratory (**A**, **C**, **E**) and field (**B**, **D**, **F**) conditions. Laboratory treatments are shown as red (20 °C experiment) and blue (6 °C experiment). Field deployments occurred in summer (orange) and winter (light blue). Smoothers are loess smoothers with a span of 0.5 and error envelopes show standard error for the loess fit. Circle symbols show individual polyp values, boxes indicate median and inter-quartile range. For reference, the mean *δ*^15^N and *δ*^13^C isotopic compositions of *Artemia* nauplii were 13.79 ± 0.86‰, and − 21.71 ± 0.30‰, respectively

At 20 °C, the mean dry weight of *Aurelia aurita* polyps decreased by 41% after 42 d (Two-way ANOVA, F(1,16) = 7.04, *P* < 0.05). At 6 °C, the mean dry weight was similar, with a 4% increase over the experiment (Fig. 3). Averaged CN (molar) ratios were around 5.7, but increased particularly between days 21 and 35, and especially in the 20 °C treatment (Fig. 4).

Isotopic values of nitrogen differed significantly between polyps maintained in the laboratory at 6 and 20 °C, respectively, with an increase of *δ*^15^N values by 1 ‰ in polyps raised at 20 °C after 21 days (GAM, *F* = 84.55, *P* < 0.0001, Table 1 and Fig. 4). At 6 °C, *δ*^15^N values of lab polyps initially decreased by 1.5 ‰ but did not change significantly over the remaining time (42 days) (GAM, *F* = 0.91, *P* > 0.05). *δ*^13^C values decreased by 1.5 ‰ over time (GAM, *F* = 44.80, *P* < 0.0001), but *δ*^13^C values did not differ in polyps maintained at 6 and 20 °C (GAM, *F* = 3.47, *P* > 0.05) (Table 2 and Fig. 4). The mean (± SD) isotopic composition of *Artemia* was 13.79 ± 0.86 ‰ for *δ*^15^N values and − 21.71 ± 0.30 ‰ for *δ*^13^C values (*n* = 13).

**Table 1:** Results of the generalised additive model (GAM)

	*Df*	*Sum Sq*	*Mean Sq*	*F value*	*Pr (*> *F)*
Time	1	0.272	0.272	0.907	0.3440
Temperature	1	25.307	25.307	84.554	< 0.0001 ***
Time:Temperature	1	6.481	6.481	21.654	< 0.0001 ***
Residuals	77	23.046	0.299		

The effect of temperature and the interaction between time and temperature were significant on the *δ*^15^N isotopic composition of laboratory polyps

Levels of significance: *P* < 0.001 ***; < 0.01 **; < 0.05 *

*Df* Degrees of freedom; *Sum Sq* Sum of squares; *Mean Sq* Mean square

**Table 2:** Results of the generalised additive model (GAM)

	*Df*	*Sum Sq*	*Mean Sq*	*F value*	*Pr (*> *F)*
Time	1	4.870	4.870	44.798	< 0.0001***
Temperature	1	0.377	0.377	3.467	0.066
Residuals	78	8.479	0.109		

The effect of time was significant on the *δ*^13^C isotopic composition of laboratory polyps

Levels of significance: *P* < 0.001 ***; < 0.01 **; < 0.05 *

*Df* Degrees of freedom; *Sum Sq* Sum of squares; *Mean Sq* Mean square

### Field experiments

**Fig. 5:**
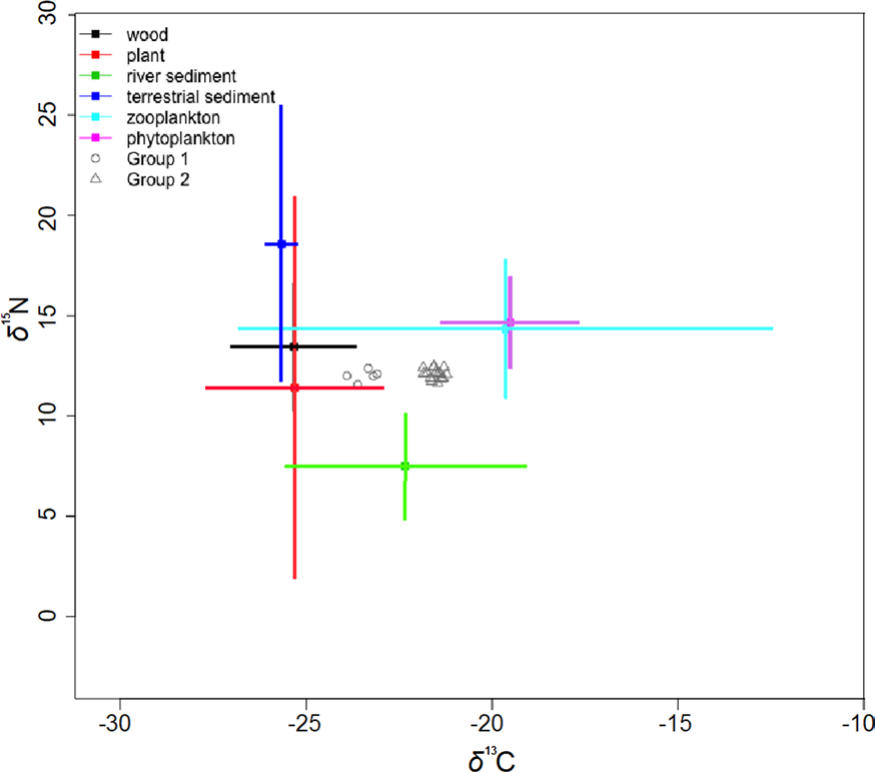
Biplot of *δ* ^15^N and *δ* ^13^C in *Aurelia aurita* polyps kept in the field (Beaulieu River) during winter (Group 1) and summer (Group 2) along with their potential food sources including plant, wood litter, sediment from the river and saltmarsh, phytoplankton and zooplankton

The mean ± standard deviation (SD) isotopic compositions of nitrogen (N) and carbon (C) in potential nutrient sources from the Beaulieu River differed between pelagic sources: phytoplankton (N 14.66 ± 1.58 ‰/C − 19.53 ± 3.56 ‰) and zooplankton (N 14.32 ± 4.24 ‰/C − 19.62 ± 7.27 ‰), with detrital sources including sediment (N 18.55 ± 7.80 ‰/C − 25.66 ± 0.21 ‰), wood litter (N 13.43 ± 3.72 ‰/C − 25.33 ± 3.28 ‰), plant (N 11.39 ± 9.91 ‰/C − 25.31 ± 4.67 ‰), and river sediment (N 7.48 ± 2.50 ‰/C − 22.34 ± 4.33 ‰) (Fig. 5). Terrestrial plant debris was consistently depleted in *δ*^13^C, reflecting exclusively C-3 vegetation sources. Sediment from the river showed *δ*^13^C values midway between aquatic and detrital plants consistent with a mixed contribution of aquatic and terrestrial sources to the detrital organic pool. Mean *δ*^13^C values of polyps differed between summer (− 21.90 ± 0.38 ‰) and winter (− 23.42 ± 0.33 ‰), whereas mean *δ*^15^N of polyps were similar in summer and winter (Fig. 5).

### Temporal variability in field polyps stable isotope values

*δ*^15^N values in field-deployed polyps changed significantly over time (GAM, *F* = 26.17, *P* < 0.0001) (Table 3 and Fig. 4). In summer, *δ*^15^N values of field-deployed polyps decreased relative to initial values by 2 ‰ over 42 days, while in winter *δ*^15^N initially decreased by about 1 ‰ then increased sharply after 21 days by 1.5 ‰ before decreasing again. Overall, *δ*^15^N values declined over time in both field experiments and were significantly lower in summer compared to winter (GAM, *F* = 85.92, *P* < 0.0001). *δ*^13^C values of field-deployed polyps decreased over 42 days (GAM, *F* = 185.65, *P* < 0.0001), with a greater decrease in winter compared to summer (GAM, *F* = 15.36, *P* < 0.0001) (Table 4 and Fig. 4). Molar CN ratios of field-deployed polyps varied simultaneously with *δ*^15^N values. In summer, CN ratios systematically decreased from an initial value of 5.6 at day 0 to 4.9 at day 42, while in winter CN ratios initially increased from 5.7 to a maximum value of 6.1 at day 7 before reducing to c. 5.3 by day 42 (Fig. 4).

**Table 3:** Results of the generalised additive model (GAM)

	*Df*	*Sum Sq*	*Mean Sq*	*F value*	*Pr (*> *F)*
Time	1	10.002	10.002	26.173	< 0.0001***
Temperature	1	13.823	13.823	85.923	< 0.0001***
Time:Temperature	1	1.654	1.654	10.281	0.002**
Residuals	65	10.457	0.161		

The effect of time, temperature and the interaction between time and temperature were significant on the isotopic composition of *δ* ^15^N of field polyps

Levels of significance: *P* < 0.001 ***; < 0.01 **; < 0.05 *

*Df* Degrees of freedom; *Sum Sq* Sum of squares; *Mean Sq* Mean square

**Table 4:** Results of the generalised additive model (GAM)

	*Df*	*Sum Sq*	*Mean Sq*	*F value*	*Pr (*> *F)*
Time	1	20.185	20.185	185.645	< 0.0001***
Temperature	1	1.670	1.670	15.358	0.0001***
Time:Temperature	1	2.370	2.370	21.800	< 0.0001***
Residuals	65	7.067	0.109		

The effect of time, temperature and the interaction between time and temperature were significant on the isotopic composition of *δ* ^13^C of field polyps

Levels of significance: *P* < 0.001 ***; < 0.01 **; < 0.05 *

*Df* Degrees of freedom; *Sum Sq* Sum of squares; *Mean Sq* Mean square

## Discussion

### Laboratory experiments

The isotopic composition and CN ratios of *Aurelia aurita* polyps measured during the laboratory experiments suggest differences in the nutritional suitability of *Artemia* under the two temperature conditions. At 6 °C, growth of polyps as indicated by weight changes over the 42 d was limited, presumably because of their low metabolic rates. CN ratios (an indication of protein content) remained relatively stable throughout the 6 °C treatment, supporting suggestions of minimal protein acquisition. Accordingly, *A. aurita* polyps held at 6 °C showed only limited isotopic shifts towards the composition of *Artemia.*

The increase in temperature between 6 and 20 °C will have caused an increase in basal metabolic rate (Gambill & Peck, 2014; Höhn et al., 2017), which must be met through metabolism of food resources. In our experiments at 20 °C, polyps showed a marked reduction in body mass over the 42-day period, coinciding with marked increases in CN ratio up to 35 days and qualitative indicators of poor health such as “softer” texture, paler colouration, and shorter tentacles. Weight loss coincided with reductions in CN ratios and increases in *δ*^15^N values are consistent with catabolism of body proteins induced by starvation. Together these observations imply that food sources provided were either indigestible, or insufficient to meet the metabolic requirements of the higher temperature.

Laboratory experiments measuring respiration in *Aurelia* polyps from several populations in northern Europe, including southern England, the Baltic Sea and Norway show that respiration rates decrease at temperatures > 15 °C (Gambill & Peck, 2014; Höhn et al., 2017), indicating that polyps likely reached their thermal limit at 20 °C in this experiment. Laboratory experiments by Kamiyama (2013) using ciliates as food for *A. aurita* polyps suggest that polyps might ingest and absorb ciliates more easily than *Artemia,* potentially explaining how polyps are able to thrive in the Beaulieu River where summer water temperatures frequently exceed 20 °C.

### Field experiments

During both the winter and summer season, polyps’ *δ*^15^N and *δ*^13^C values declined from the starting laboratory *Artemia-*raised signal. In summer CN ratios declined systematically from the initial laboratory-reared value of c. 5.6 to a final low of 4.9, while in winter, CN ratios initially increased but ultimately reduced to values lower than the initial laboratory-raised polyps, indicating ingestion and assimilation of new food sources with relatively depleted isotopic compositions. In the Beaulieu River, in situ phytoplankton, zooplankton, and saltmarsh sediment sources all were enriched in ^15^N compared to *Artemia,* and in-river sediment was the only sampled nutrient source that was depleted in ^15^N compared to *Artemia.* Similarly*,* pelagic nutrient sources represented by zooplankton and phytoplankton were enriched in ^13^C compared to *Artemia,* whereas benthic nutrient sources, with contributions from isotopically depleted terrestrial plants, were depleted in ^13^C relative to *Artemia.* The consistent negative shift in polyp *δ*^15^N and *δ*^13^C values following deployment in the field therefore implies that polyp metabolism was supported by benthic-derived nutrient pathways potentially involving sediment-inhabiting and/or epifaunal bacteria, epibenthic organisms and detritivores.

Carbon assimilated by polyps was markedly more negative in winter than in summer, suggesting that pelagic production contributes more to the polyp diet in summer. CN ratios reduced rapidly and systematically in summer, however, implying that zooplankton food sources provide higher nutritional content and promote more protein synthesis than detrital food sources available in winter. Zooplankton-dominated diet sources consumed during the summer deployment promoted rapid protein synthesis indicated by the immediate and consistent reductions in CN ratios when polyps were transferred from their *Artemia* diet, strongly implying that the laboratory diet impeded protein synthesis.

Seasonal changes in the diets of *Aurelia aurita* medusae (Javidpour et al., 2016) and *Aurelia coerulea* polyps (Pengpeng et al., 2021) have been inferred from stable isotope and gut contents analysis (GCA) studies in the Kiel Bight and Jiaozhou Bay, respectively. In the study of Javidpour et al. (2016), a benthic-pelagic dietary shift from mesozooplankton to microplankton and small, resuspended benthic particles was identified for medusae of *A. aurita* but not for *Cyanea capillata*. The GCA method used by Pengpeng et al. (2021) also revealed seasonality in the diet of *A. coerulea* polyps, but only involving changes within the planktonic prey (copepods, copepod nauplii, ciliates). It was suggested that low incidence of ciliates and nauplii in the guts of polyps might reflect rapid digestion of these prey items, thus underestimating their role in the diet. This could also apply to a lack of benthic-derived food sources noted in that study.

Our observations of a switch from pelagic to benthic-derived food sources in *A. aurita* polyps are consistent with other stable-isotope-based studies of seasonal variation in nutrient sources in temperate aquatic ecosystems. For example, in a temperate lake in the UK, Grey & Jones (2001) used seasonally sampled carbon isotope compositions to demonstrate that zooplankton obtained nutrients derived from particulate organic matter during winter and early spring, but from algal production in summer. Zooplankton taxa growing throughout the year derived nutrients from both allochthonous and *in situ* production, whereas taxa growing only in summer derived almost all carbon from in situ algal sources.

In this study, we have demonstrated that previous assumptions stating that the polyp life stage in *Aurelia* is supported exclusively by pelagic sources such as zooplankton and phytoplankton, may be an oversimplification, at least in shallow tidal estuaries. In the Beaulieu River, nutrient sources supporting polyps changed from in situ pelagic sources in summer to allochthonous benthic-derived sources in winter when pelagic plankton productivity was at its lowest. During winter, ingested carbon was incorporated into somatic growth, resulting in bigger polyps, which can then transform into strobilae consisting of a greater number of discs, thus producing more ephyrae per polyp (Wang et al., 2015). Laboratory polyps maintained on *Artemia* failed to meet their metabolic costs at 20 °C, and at 6 °C did not grow sufficiently to strobilate, presumably because they were not extracting sufficient nutrients from the *Artemia* diet. As a result, reproductive rates (strobilation) inferred from laboratory experiments where polyps are exclusively fed *Artemia* may not reflect natural rates. Predictions of cascading effects such as bloom events based on laboratory-based estimates of reproductive metabolism should be approached with caution.

## Conclusion

This study provides evidence of a difference in *Artemia* assimilation by *Aurelia aurita* polyps at two different temperatures. While at 6 °C the metabolic rate and assimilation of *Artemia* was restricted, at 20 °C the polyp metabolic cost exceeded the assimilation of *Artemia*, leading to starvation. Field-based stable isotope analyses highlighted a previously unrecognised importance of benthic-derived nutrient sources supporting *A. aurita* polyps in the Beaulieu River, particularly during winter. Temperate estuaries are highly variable and dynamic environments and are critical transition zones linking terrestrial, freshwater, and marine habitats, and their associated food sources. *Aurelia aurita* inhabits a wide range of environments, in particular temperate shallow estuaries, and the current study highlights its adaptation to seasonally available food sources. Generalist populations with broader niches are likely favoured in heterogeneous and stable environments (e.g. benthic ecosystems). Finally, this study implies that laboratory-based observations of physiological rates or ecological behaviours in polyps that are nourished exclusively by *Artemia* may not be representative of wild populations.

## Data Availability

The datasets used and/or analysed during the current study are available from the corresponding author on reasonable request. The authors confirm that the data supporting the findings of this study are available within the article.
